# Osteoblast Response of Additively Manufactured Zirconia and Alumina-Toughened Zirconia

**DOI:** 10.3390/ma15238685

**Published:** 2022-12-06

**Authors:** Hiroto Nakai, Masanao Inokoshi, Kosuke Nozaki, Kumiko Yoshihara, Akihiro Matsukawa, Noriyuki Nagaoka, Watcharapong Tonprasong, Shunsuke Minakuchi

**Affiliations:** 1 Department of Gerodontology and Oral Rehabilitation, Graduate School of Medical and Dental Sciences, Tokyo Medical and Dental University, 1-5-45 Yushima, Bunkyo, Tokyo 113-8549, Japan; 2 Department of Advanced Prosthodontics, Graduate School of Medical and Dental Sciences, Tokyo Medical and Dental University, 1-5-45 Yushima, Bunkyo, Tokyo 113-8549, Japan; 3 Health and Medical Research Institute, National Institute of Advanced Industrial Science and Technology (AIST), 2217-14 Hayashicho, Takamatsu 761-0395, Japan; 4 Department of Pathology & Experimental Medicine, Graduate School of Medicine, Dentistry and Pharmaceutical Sciences, Okayama University, 2-5-1 Shikatacho, Kita, Okayama 700-8558, Japan; 5 Advanced Research Center for Oral and Craniofacial Sciences, Okayama University Dental School, 2-5-1 Shikatacho, Kita, Okayama 700-8558, Japan; 6 Department of Restorative Dentistry, Faculty of Dentistry, Naresuan University, 99 Village No.9, Phitsanulok-Nakhon Sawan Road, Tha Pho, Mueang Phitsanulok District, Phitsanulok 65000, Thailand

**Keywords:** zirconia, additive manufacturing, alumina-toughened zirconia, cell viability, osteogenic ability

## Abstract

Zirconia ceramics have been widely used in dentistry. Herein, we assess the surface morphology, surface texture, and osteoblast response of additively manufactured zirconia and alumina-toughened zirconia (ATZ) in comparison with titanium. The surface roughness, contact angle, and surface microstructure of titanium sandblasted with large-grit alumina and subsequently acid-etched using 18% HCl and 49% H_2_SO_4_ (SLA-titanium), uniaxially pressed zirconia (UP zirconia), additively manufactured zirconia (AM zirconia), and additively manufactured ATZ (AM ATZ) were investigated. Moreover, the cell viability, alkaline phosphatase (ALP) activity, and gene expression of type I collagen on these materials were evaluated. The data were statistically analyzed using one-way ANOVA with Tukey’s post hoc test. SLA-titanium showed the highest surface roughness and contact angle. The other three materials showed comparable surface roughness and contact angles. Micro- and nanoroughness were observed on the surface of SLA-titanium. UP zirconia and AM zirconia had similar surface morphologies. The cell viability, ALP activity, and gene expression of type I collagen on AM zirconia were comparable to or better than those on SLA-titanium. Our results indicate that AM zirconia is a promising material for zirconia dental implants.

## 1. Introduction

Zirconia ceramics have been widely used as alternatives to metals in dental restorations owing to their biocompatibility, mechanical properties, and aesthetics [1]. Nowadays, zirconia ceramics are used not only in dental prostheses such as crowns and bridges but also in dental implant abutments and fixtures [2].

A recent position paper reported that one-piece zirconia implants show comparable clinical prognoses to conventional titanium implants [3]. Currently, commercially available zirconia implants are fabricated via the following process: (1) given an implant body shape, zirconia powder is filled into a mold; it is then subjected to (2) cold isostatic pressing using water pressure, (3) sintering, and (4) machining [4,5]. To ensure sufficient osseointegration, zirconia implants need surface treatments such as sandblasting, laser irradiation, and acid etching after sintering and machining. In general, conventional zirconia implants sandblasted with alumina particles followed by acid-etching with hydrofluoric acid have similar bioactivity to titanium implants [6]. However, these surface treatments may damage the zirconia implant surface and adversely affect its mechanical properties [7,8,9]. The fabrication of zirconia implants via additive manufacturing can avoid these issues because complex surface structures can be created without requiring additional surface treatments.

In a previous study, we clarified the basic properties of additively manufactured zirconia ceramics [10]. Our results indicated that additively manufactured 3 mol% yttria-stabilized tetragonal zirconia polycrystals (3Y-TZP) and additively manufactured alumina-toughened zirconia (ATZ) have desirable mechanical properties and crystal structures comparable to those of subtractively manufactured dental zirconia. Moreover, Gong et al. reported that uniaxially pressed 3Y-TZP had no harmful effects on cell proliferation and no genetic problems during bone formation [11]. However, the osteoblast response of additively manufactured zirconia is not yet fully understood. In particular, the influence of surface morphology and surface texture on the osteoblast response of additively manufactured zirconia and ATZ is not clear.

Therefore, this study aimed to assess the influence of surface morphology and surface texture on the osteoblast response of additively manufactured zirconia and ATZ in comparison with that of titanium. The null hypothesis was that the surface morphology, surface texture, and osteoblast response of additively manufactured zirconia and ATZ are similar to those of titanium.

## 2. Materials and Methods

### 2.1. Specimen Preparation

The following four materials were investigated: (1) sandblast with large-grit and acid-etched titanium (SLA-titanium; Rare Metallic Co., Ltd., Tokyo, Japan): the titanium specimen was polished with #320 abrasive paper and sandblasted with large-grit alumina (φ 250 µm), followed by acid-etching using 18% HCl and 49% H_2_SO_4_ at 60 °C for 30 min; (2) uniaxially pressed 3Y-TZP (UP zirconia; TZ-3YSB-E, Tosoh, Tokyo, Japan); (3) additively manufactured zirconia (AM zirconia; 3D Mix zirconia, 3DCeram Sinto, Limoges, France): specimens were prepared using a stereolithography system (CERAMAKER C900, 3DCeram Sinto) with a build direction of 0°; and (4) additively manufactured ATZ (AM ATZ; 3D Mix ATZ, 3DCeram Sinto): specimens were prepared using a stereolithography system (CERAMAKER C900, 3DCeram Sinto) with a build direction of 0°. Except for SLA-titanium, all specimens were used in the as-sintered conditions without any surface treatments. All specimens were prepared in the shape of a disk with a diameter of 10.8 mm and thickness of 1.2 mm (n = 27/group).

### 2.2. Surface Roughness

The surface roughness was examined via three-dimensional (3D) confocal laser microscopy (LEXT4100, Olympus, Tokyo, Japan). Ten regions (the effective field of view was 256 µm) were selected for each specimen. The average values of S_a_ (arithmetic mean height) and R_a_ (arithmetic mean roughness) were calculated using ProfilmOnline software (https://www.profilmonline.com/ (accessed on 5 November 2022), Filmetrics, San Diego, CA, USA) with an 80 μm cutoff value.

### 2.3. Surface Wettability

The contact angle was measured to assess the wettability of each specimen. A 2 µL droplet of distilled water was placed on the sample surface, and the contact angle was measured using an automatic contact angle meter (SImage AUTO 100, Excimer, Kanagawa, Japan).

### 2.4. Microstructural Analysis Using a Scanning Electron Microscope

Microstructural analyses were conducted using scanning electron microscopy (SEM). Before the observation, the specimens were coated with osmium using an osmium coater (Neoc-STB, Meiwafosis Co., Ltd., Tokyo, Japan). The coated specimens were observed using a field-emission-gun SEM (FEG-SEM; SU8230, Hitachi, Tokyo, Japan) operated at 5 kV.

### 2.5. Cell Viability

Mouse osteoblast-like cells (MC3T3-E1 cells, RIKEN BRC, Tsukuba, Japan) were used to assess the cell viability of each specimen [12]. Cells were cultured at 37 °C in a 5% CO_2_ incubator using an alpha modification of Eagle’s minimal essential medium (α-MEM, Nacalai Tesque, Kyoto, Japan). Fetal bovine serum (FBS) and 1% penicillin-streptomycin (PS) solution were added to this medium. The culture medium was replaced every 2 or 3 days. The four specimens (SLA-titanium, UP zirconia, AM zirconia, and AM ATZ) were cleaned with ethanol and distilled water and then dried. The specimens were sterilized in an autoclave (Autoclave SX-300, TOMY Seiko Co., Ltd., Tokyo, Japan) for 20 min at 121 °C and placed on a 24-well plate. MC3T3-E1 cells cultured in the aforementioned conditions were seeded at a density of 5 × 10^4^ cells/mL on each disk. Cells were reacted using a Cell Proliferation Kit I (3-(4,5-dimethyl-2-thiazolyl)-2,5-diphenyltetrazolium bromide (MTT); Roche, Sigma-Aldrich, St. Louis, MO, USA) after 1, 3, and 7 days. The standard solution was prepared and reacted for 30 min following the protocol provided by the manufacturer (Roche, Sigma-Aldrich). The absorbance was measured using a spectrophotometer at 550 nm.

### 2.6. Alkaline Phosphatase (ALP) Activity

MC3T3-E1 cells (RIKEN BRC) were cultured at 37 °C in a 5% CO_2_ incubator. α-MEM (FUJIFILM Wako Shibayagi Corporation, Gunma, Japan) with 10% FBS (Thermo Fisher Scientific, Waltham, MA, USA) and 1% PS solution (FUJIFILM Wako Shibayagi Corporation) was used and exchanged every 2 or 3 days. The four specimens were cleaned with 70% ethanol and distilled water and then dried. They were then placed in a 48-well plate. MC3T3-E1 cells cultured in the aforementioned conditions were seeded at a density of 5 × 10^4^ cells/mL on each specimen. α-MEM with 10% FBS and 1% PS solution was supplemented with 50 μg/mL ascorbic acid (Sigma-Aldrich), 10 mM β-glycerophosphate (Sigma-Aldrich), and 1 µM dexamethasone (Sigma-Aldrich) and was used as a medium for inducing differentiation. The supernatant was removed, and the cells were washed with sterilized phosphate-buffered saline (PBS) before cell fixation. The cells were fixed using 4% paraformaldehyde (PFA) and then washed twice with PBS. The cells were frozen by cooling from room temperature (20–25 °C) to −20 °C in TritonX-100 (Nacalai Tesque) and then melted by heating from −20 °C to room temperature (20–25 °C) in TritonX-100 three times. The alkaline phosphatase (ALP) activity and total amount of protein were quantified using LabAssay™ ALP (FUJIFILM Wako Shibayagi Corporation) and a BCA™ Protein Assay Kit (Thermo Fisher Scientific), respectively. The absorbance was measured at 405 and 562 nm and the ALP activity and amount of protein were analyzed.

### 2.7. Analysis of Gene Expression by Real-Time PCR

MC3T3-E1 cells (RIKEN BRC) were cultured for 7 and 14 days in a manner similar to that described in Section 2.5. The RNeasy Micro Kit (Qiagen, Venlo, The Netherlands) was used to extract mRNA from the cells on each specimen. Reverse transcript cDNA was obtained using High Capacity cDNA Reverse Transcription Kits (Thermo Fisher Scientific) [13,14]. Type I collagen was used as the primer, and glyceraldehyde 3 phosphate dehydrogenase was selected as an endogenous control. The gene expression was quantified using Applied Biosystems^®^ StepOne™ (Thermo Fisher Scientific).

### 2.8. Statistical Analysis

The results of the contact angle, cell viability, ALP activity, and real-time PCR tests were statistically analyzed using the Shapiro–Wilk test followed by either one-way analysis of variance (ANOVA) and Tukey’s post hoc test or the Kruskal–Wallis test and Dunn test. Moreover, the Pearson correlation coefficients were calculated to clarify the relationship between 11 factors: surface roughness (S_a_, R_a_), contact angle, cell viability (at 1, 3, and 7 days), ALP activity (at 7, 10, and 14 days), and gene expression of type I collagen (at 7 and 14 days). All tests were performed at a significance level of α = 0.05 using a statistical software package (R4.2.0, R Foundation for Statistical Computing, Vienna, Austria).

## 3. Results

### 3.1. Surface Roughness

The results of the surface roughness analysis are shown in Table 1. S_a_ and R_a_ of titanium were higher than those of the other three specimens. S_a_ and R_a_ of UP zirconia, AM zirconia, and AM ATZ were similar.

### 3.2. Contact Angle

**Figure 1:**
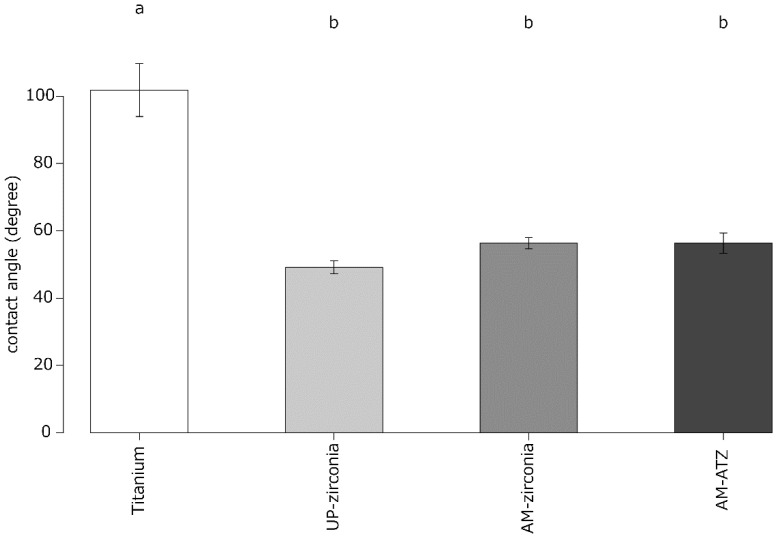
Results of contact angle measurements. Identical letters indicate no statistical difference.

The results of the contact angle measurements are shown in Figure 1. One-way ANOVA followed by Tukey’s post hoc test indicated that the contact angle of titanium was significantly higher than those of the other three grades.

### 3.3. Microstructural Analysis Using SEM

**Figure 2:**
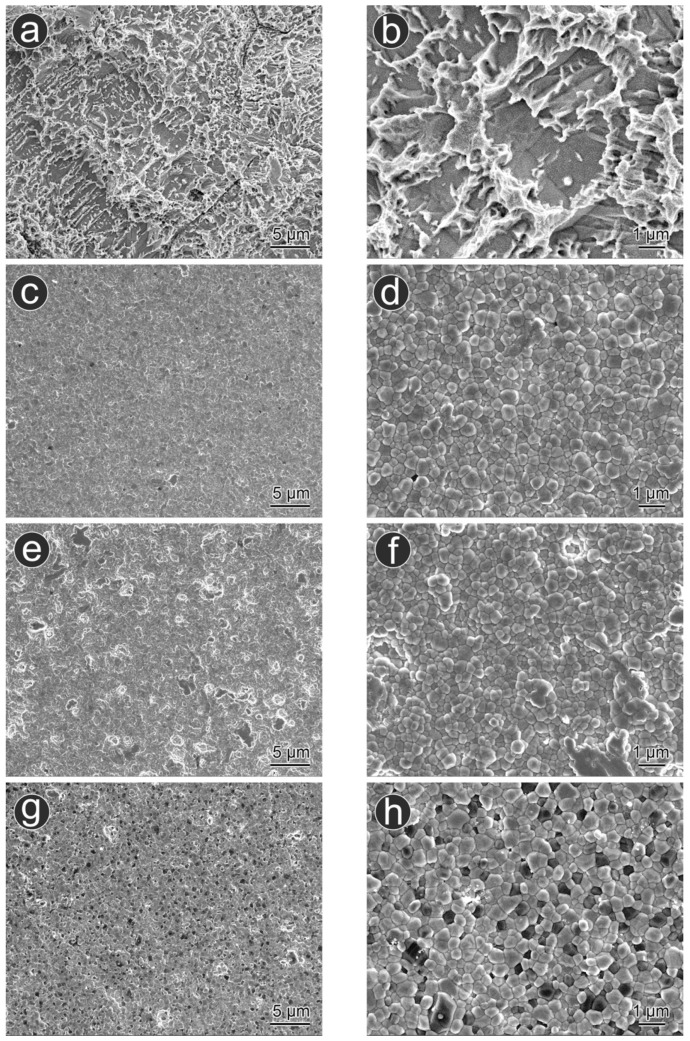
Representative scanning electron microscopy (SEM) images: (**a**,**b**) sandblasted (large-grit) and acid-etched titanium (SLA-titanium), (**c**,**d**) uniaxially pressed zirconia (UP zirconia), (**e**,**f**) additively manufactured zirconia (AM zirconia), and (**g**,**h**) additively manufactured ATZ (AM ATZ).

The results of the microstructural analysis using SEM are shown in Figure 2. The titanium surface had an irregular, needle-shaped microstructure and a rough surface due to sandblasting and nanoroughness due to acid etching (Figure 2a,b). In contrast, zirconia grains with a grain size of 1 µm or smaller and clear boundaries were observed for UP zirconia and AM zirconia (Figure 2c–f). Zirconia grains and alumina grains with a grain size of 1 µm or smaller were observed on the AM ATZ surface (Figure 2g,h).

### 3.4. Cell Viability

**Figure 3:**
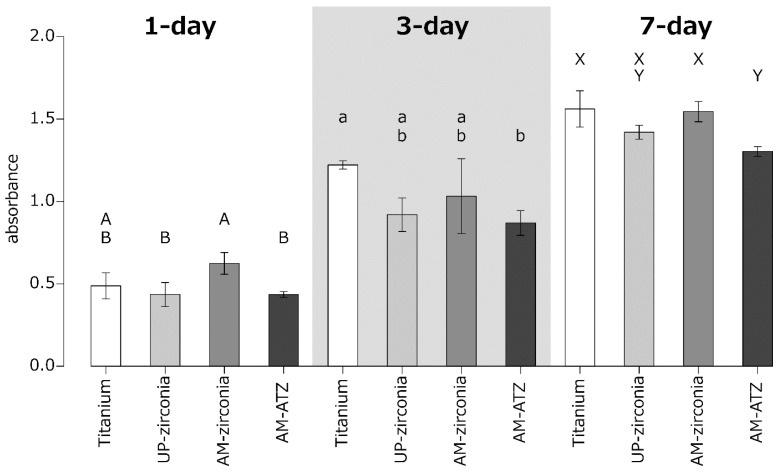
Viability of MC3T3E-1 cells on each material at 1, 3, and 7 days. Identical letters indicate no statistical difference.

The results of the cell viability analysis are shown in Figure 3. One-way ANOVA followed by Tukey’s post hoc test revealed that the cell viability on AM zirconia was significantly higher than that on AM ATZ at 1 day. In contrast, no significant difference was observed in the cell viabilities on titanium, UP zirconia, and AM zirconia at 3 days. The cell viabilities on titanium and AM zirconia were significantly higher than that on AM ATZ at 7 days. Overall, the cell viability on AM ATZ was the lowest at 1, 3, and 7 days.

### 3.5. ALP Activity

**Figure 4:**
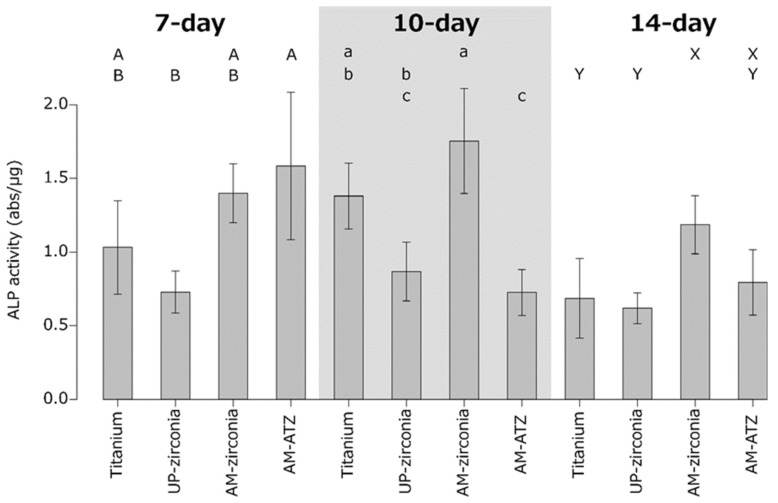
Alkaline phosphatase (ALP) activity of MC3T3E-1 cells on each material at 7, 10, and 14 days. Identical letters indicate no statistical difference.

The results of the ALP activity analysis are shown in Figure 4. One-way ANOVA followed by Tukey’s post hoc test revealed no statistically significant differences between SLA-titanium, AM zirconia, and AM ATZ at 7 days. The ALP activity of AM zirconia was comparable to that of SLA-titanium but significantly higher than those of UP zirconia and AM ATZ at 10 days. The ALP activity of AM zirconia was significantly higher than that of SLA-titanium and UP zirconia at 14 days.

### 3.6. Analysis of Gene Expression by Real-Time PCR

**Figure 5:**
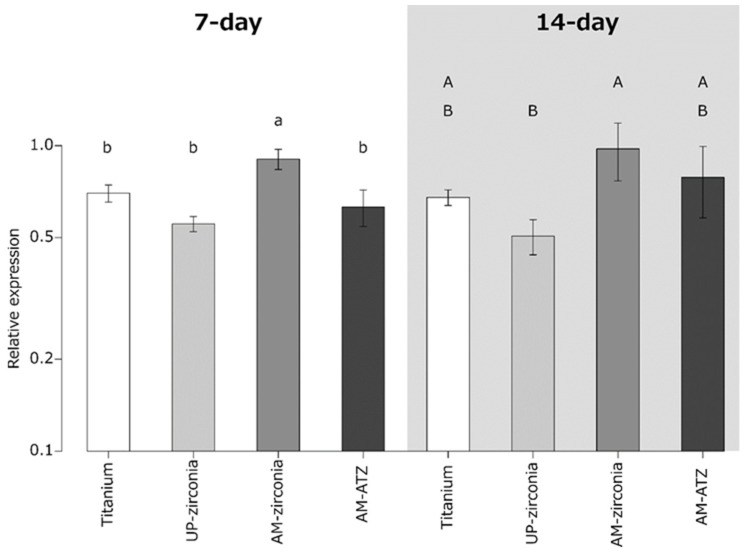
Gene expression of type 1 collagen on each material at 7 and 14 days. Identical letters indicate no statistical difference.

The results of gene expression of type I collagen are shown in Figure 5. One-way ANOVA followed by Tukey’s post hoc test revealed that the gene expression of type I collagen at 7 days was highest on AM zirconia. Moreover, the gene expression of type I collagen at 14 days was significantly higher on AM zirconia than on UP zirconia.

### 3.7. Correlation Analysis

**Figure 6:**
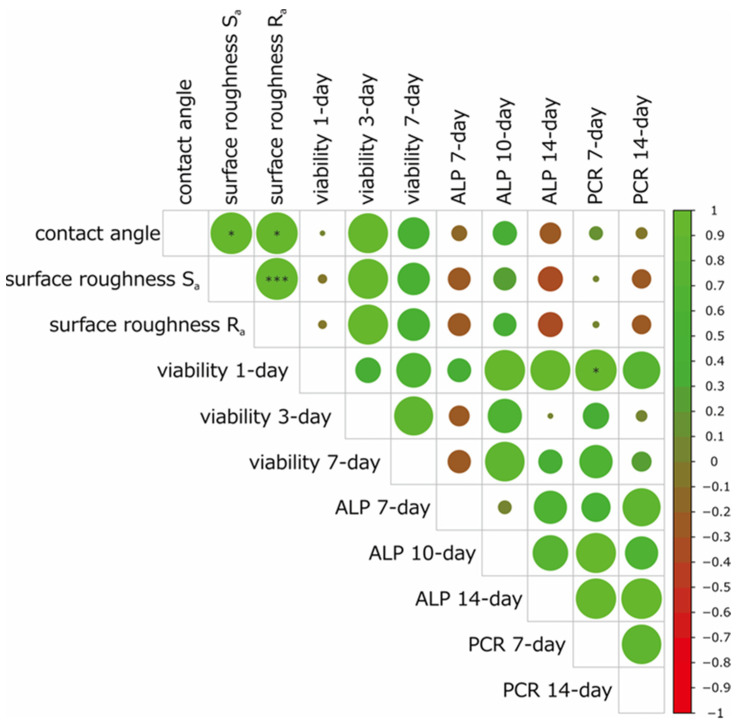
Results of the Pearson’s correlation analysis of each factor. Green and red circles denote positive and negative correlations, respectively. * α < 0.05; *** α < 0.001.

A summary of the correlation analysis is presented in Figure 6. The contact angle was positively correlated with the surface roughness (S_a_ and R_a_), cell viability at 3 and 7 days, and ALP activity at 10 days. S_a_ and R_a_ were positively correlated with the cell viability at 3 and 7 days and ALP activity at 10 days. The cell viability at 1 day was positively correlated with the gene expression of RT-PCR at 7 days. The cell viabilities at 3 and 7 days were positively correlated with the ALP activity at 10 days. The gene expression of RT-PCR at 7 days was positively correlated with the gene expression of RT-PCR at 14 days. In contrast, the ALP activity at 7 days was negatively correlated with the contact angle, surface roughness (S_a_ and R_a_) and cell viability at 3 and 7 days.

## 4. Discussion

The results of the present study indicate that the surface morphologies and surface textures of the four materials investigated herein were different. Moreover, the cell viability of AM zirconia was comparable to that of SLA-titanium at 1, 3, and 7 days. However, the cell viability of AM ATZ was significantly lower than that of SLA-titanium at 3 and 7 days. The ALP activity of AM zirconia was significantly higher than that of UP zirconia. The gene expression of type I collagen on AM zirconia was highest at both 7 and 14 days. Thus, the null hypothesis ‘surface morphology, surface texture, and osteoblast response of additively manufactured zirconia and ATZ are similar to those of titanium’ was partially rejected.

Currently, few studies have investigated the cell viability and osteoblast response of AM zirconia. SLA-titanium was selected as a control because it has excellent osteogenic ability [11]. Zirconia specimens fabricated via molding with uniaxial pressure were included as well because they were considered to be the same as commercially available dental zirconia implants. To eliminate the influence of surface treatments, the specimens herein—except SLA-titanium—were not subjected to any surface treatments.

In the present study, an MTT assay was used to assess the cell viability. When MTT is introduced into the cells, dehydrogenase present in the mitochondria deoxidizes MTT into formazan dye. The amount of formazan dye is correlated with the cell population that shows metabolism, and the living cell population is measured using the MTT assay. The MC3T3-E1 cells used for cell culture on each specimen express ALP upon differentiation. The ALP activity was assessed because it is a differentiation marker of osteoblasts [12]. In addition, type I collagen is a major component of the extracellular matrix and is important for the calcification of osteoblasts [15]. Therefore, the gene expression of type I collagen was investigated as a marker of osteogenic ability. Moreover, in the present study, we sterilized specimens with an autoclave to remove contamination. However, it should be noted that sterilization in an autoclave may affect the specimen biocompatibility [16].

In the present study, SLA-titanium presented the roughest surface. Strickstrock et al. reported that the S_a_ of SLA-titanium, measured by stereo-SEM, was 1.16 µm, whereas that of zirconia without surface treatment was 0.36 µm [17]. Kim et al. reported that the S_a_ of SLA-titanium was 0.65 µm, whereas that of as-sintered AM zirconia was 0.54 µm [18]. Buser et al. reported that the S_a_ of SLA-titanium was 1.15 µm [19], and Zhao et al. reported that the S_a_ of SLA-titanium was 1.2 µm [20]. The S_a_ of SLA-titanium in the present study was 2.23 µm, which is higher than those reported previously [17,18,19,20]. However, the S_a_ of AM zirconia in the present study was 0.27 µm, which is similar to those reported previously [17,18]. The influence of the surface quality of titanium on bone response has been investigated in a systematic review [21]: the bone responses on titanium with smooth surfaces (S_a_ < 0.5 µm) and minimally rough surfaces (S_a_ = 0.5–1.0 µm) were weaker than those on titanium with rough surfaces. In contrast, titanium with moderately rough surfaces (S_a_ = 1–2 µm) showed a stronger bone response than that with rough surfaces (S_a_ > 2 µm). Further investigations are needed to clarify the influence of surface morphology of AM zirconia on the osteogenic ability of osteoblasts.

The contact angle determines the wettability of materials. In general, a hydrophilic surface results in a better osteogenic ability than a hydrophobic surface [22]. In the present study, the average contact angle of SLA-titanium was 101.8°, which indicates that SLA-titanium can be considered hydrophobic. Schünemann et al. reported that SLA-titanium had a contact angle of more than 90° and was therefore considered hydrophobic, whereas as-sintered 3Y-TZP had a contact angle of less than 90° and was hence considered hydrophilic [23]. Gittens et al. reported that the contact angle of SLA-titanium was 157° ± 3° and considered it to be hydrophobic [24]. The results of the present study agree well with the previous reports. In the future, it would be interesting to analyze the dynamic hysteresis of different measuring droplets, such as simulated body fluid (SBF) or Ringer’s solution, to assess another aspect of the fluid mechanics of the surfaces.

Jakovac et al. reported that 3Y-TZP grains had clear grain boundaries and a grain size of 1 µm or smaller [25]. The SEM images of UP zirconia and AM zirconia in the present study were similar to those of Jakovac et al. [25]. Kim et al. reported that SLA-titanium (sandblasted with 250–500 µm alumina particles) showed microroughness due to sandblasting and nanoroughness due to acid etching [26]. Our results agree well with their report. Moreover, Mario et al. reported that dense grains with clear grain boundaries were observed on AM zirconia [27]. They also reported that the microstructure of AM ATZ was different from that of AM zirconia because the former contains a significant number of alumina grains [27]. The SEM images of AM ATZ in the present study were comparable to those of previous studies [27].

The cell viability on SLA-titanium and AM zirconia—analyzed using MTT assay—was higher than that on AM ATZ. The surface roughness (S_a_, R_a_) and contact angle of AM ATZ were lower than those of titanium, whereas they were comparable with those of AM zirconia. Because the surface roughness (S_a_, R_a_) and contact angles of AM zirconia and AM ATZ were comparable, we suggest that the difference in the composition of AM zirconia and AM ATZ (specifically the alumina content; AM zirconia and AM ATZ contain ~0.05 and ~20 wt% alumina, respectively [10]) might affect their cell viability.

AM zirconia presented significantly higher ALP activity than UP zirconia at 10 and 14 days. Ito et al. compared the ALP activity of four differently treated zirconia materials: mirror-polished zirconia (MS), zirconia sandblasted with 50 µm alumina (SB 50), zirconia blasted with 150 µm alumina (SB 150), and zirconia blasted with 150 µm alumina and acid-etched with hydrofluoric acid (SB 150E). Their results showed that the ALP activities of SB 50, SB 150, and SB 150E were higher than that of MS at 7 days [6]. Hirano et al. also reported that the ALP activity of SB 150E was significantly higher than that of MS at 7 days [28]. Their results indicated that rougher surfaces present higher ALP activities. In this study, although SLA-titanium had the highest surface roughness, it did not show the highest ALP activity. This was possibly because of the surface hydrophobicity of the SLA-titanium. The surface of SLA-titanium in this study might be too rough for osteoblasts because the S_a_ was 2.23 µm, which is rougher than the moderately rough surface reported previously (S_a_ = 1–2 µm) [21].

In the present study, no significant difference in the gene expression of type I collagen was observed between SLA-titanium and UP zirconia. However, the gene expression of type I collagen on AM zirconia was significantly higher than that on the other materials at 7 days and significantly higher than that on UP zirconia at 14 days. Ito et al. reported that there were no significant differences in the gene expression of type I collagen among MS, SB 50, SB 150, and SB 150E groups at 3, 7, and 14 days [6]. Rohr et al. reported that no significant differences were detected in the gene expression of type I collagen between as-sintered zirconia and SLA-zirconia [5]. Our results are in good agreement with the previous reports. Although the compositions of UP zirconia and AM zirconia are comparable, a significant difference in the gene expression of type I collagen was observed. This difference might be related to the surface morphology. Further investigations are needed to clarify other gene expressions such as osteocalcin and osteopontin.

The correlation analysis revealed a strong positive correlation between the surface roughness (S_a_, R_a_) and contact angle. In general, a higher surface roughness results in a lower contact angle for hydrophilic materials, whereas a higher surface roughness results in a higher contact angle for hydrophobic materials [29]. In accordance with this principle, SLA-titanium had the highest surface roughness and highest contact angle in the present study. The ALP activity at 7 days was negatively correlated with the surface roughness (S_a_, R_a_) and contact angle. Nishimura et al. reported that a hydrophilic surface was more advantageous to initial cell attachment than a hydrophobic surface [22]. It is therefore reasonable that SLA-titanium showed lower ALP activity at 7 days.

In this study, AM zirconia presented acceptable cell viability and gene expression of type I collagen. Zirconia ceramics show osteogenic ability not only in vitro but also in vivo. Uchida et al. reported that zirconia with tetragonal or monoclinic phases exhibited high apatite-forming ability in SBF [30]. Li et al. reported that the ALP activity and gene expression of osteopontin were higher on zirconia with microgrooves ablated using a femtosecond laser than on mirror-polished zirconia [31]. AM zirconia can be fabricated with a complex surface morphology within the resolution of a 3D printer without any negative influences [32]. Further studies are needed to clarify the optimal surface morphologies for improving the cell viability, ALP activity, and gene expression on AM zirconia with various microstructures. Moreover, in vivo animal studies are needed to examine the actual bone response of AM zirconia implants.

## 5. Conclusions

The cell viability, ALP activity, and gene expression of type I collagen of AM zirconia were comparable to those of SLA-titanium. Thus, AM zirconia is considered suitable for applications in dental implants. In contrast, the cell viability of AM ATZ was lower than that of SLA-titanium. The results of the present study indicate that AM zirconia is a potential candidate for zirconia dental implants.

## Figures and Tables

**Table 1:** Results of the surface roughness analysis: arithmetic mean height (S_a_) and arithmetic mean roughness (R_a_).

Material ^1^	S_a_ (µm)	R_a_ (µm)
SLA-titanium	2.23	1.69
UP zirconia	0.27	0.23
AM zirconia	0.28	0.23
AM ATZ	0.22	0.17

^1^ SLA-titanium: sandblasted (large-grit) and acid-etched titanium; UP zirconia: uniaxially pressed zirconia; AM zirconia: additively manufactured zirconia; AM ATZ: additively manufactured alumina-toughened zirconia.

## Data Availability

The data presented in this study are available from the corresponding author, M.I., upon reasonable request.

## References

[1] Saridag S., Tak O., Alniacik G. (2013). Basic properties and types of zirconia: An overview. World J. Stomatol..

[2] Roehling S., Schlegel K.A., Woelfler H., Gahlert M. (2018). Performance and outcome of zirconia dental implants in clinical studies: A meta-analysis. Clin. Oral. Implant. Res..

[3] Balmer M., Payer M., Kohal R.-J., Spies B.C. (2022). EAO position paper: Current level of evidence regarding zirconia implants in clinical trials. Int. J. Prosthodont..

[4] Grech J., Antunes E. (2019). Zirconia in dental prosthetics: A literature review. J. Mater. Res. Technol..

[5] Rohr N., Bergemann C., Nebe J.B., Fischer J. (2020). Crystal structure of zirconia affects osteoblast behavior. Dent. Mater..

[6] Ito H., Sasaki H., Saito K., Honma S., Yajima Y., Yoshinari M. (2013). Response of osteoblast-like cells to zirconia with different surface topography. Dent. Mater. J..

[7] Iijima T., Homma S., Sekine H., Sasaki H., Yajima Y., Yoshinari M. (2013). Influence of surface treatment of yttria-stabilized tetragonal zirconia polycrystal with hot isostatic pressing on cyclic fatigue strength. Dent. Mater. J..

[8] Xie H., Shen S., Qian M., Zhang F., Chen C., Tay F.R. (2015). Effects of acid treatment on dental zirconia: An in vitro study. PLoS ONE.

[9] Inokoshi M., Shimizubata M., Nozaki K., Takagaki T., Yoshihara K., Minakuchi S., Vleugels J., Van Meerbeek B., Zhang F. (2021). Impact of sandblasting on the flexural strength of highly translucent zirconia. J. Mech. Behav. Biomed. Mater..

[10] Nakai H., Inokoshi M., Nozaki K., Komatsu K., Kamijo S., Liu H., Shimizubata M., Minakuchi S., Van Meerbeek B., Vleugels J. (2021). Additively manufactured zirconia for dental applications. Materials.

[11] Gong S.-H., Lee H., Pae A., Noh K., Shin Y.-M., Lee J.-H., Woo Y.-H. (2013). Gene expression of MC3T3-E1 osteoblastic cells on titanium and zirconia surface. J. Adv. Prosthodont..

[12] Gapski R., Martinez E.F. (2017). Behavior of MC3T3-E1 osteoblastic cells cultured on titanium and zirconia surfaces: An in vitro study. Implant Dent..

[13] Gutiérrez-Aguirre I., Mehle N., Delić D., Gruden K., Mumford R., Ravnikar M. (2009). Real-time quantitative PCR based sensitive detection and genotype discrimination of *Pepino mosaic virus*. J. Virol. Methods.

[14] Schneider R.K., Puellen A., Kramann R., Raupach K., Bornemann J., Knuechel R., Pérez-Bouza A., Neuss S. (2010). The osteogenic differentiation of adult bone marrow and perinatal umbilical mesenchymal stem cells and matrix remodelling in three-dimensional collagen scaffolds. Biomaterials.

[15] Boskey A.L. (1998). Biomineralization: Conflicts, challenges, and opportunities. J. Cell Biochem..

[16] Vezeau P.J., Koorbusch G.F., Draughn R.A., Keller J.C. (1996). Effects of multiple sterilization on surface characteristics and in vitro biologic responses to titanium. J. Oral Maxillofac. Surg..

[17] Strickstrock M., Rothe H., Grohmann S., Hildebrand G., Zylla I.M., Liefeith K. (2017). Influence of surface roughness of dental zirconia implants on their mechanical stability, cell behavior and osseointegration. BioNanoMaterials.

[18] Kim J.-C., Yeo I.-S.L. (2021). Bone response to conventional titanium implants and new zirconia implants produced by additive manufacturing. Materials.

[19] Buser D., Broggini N., Wieland M., Schenk R.K., Denzer A.J., Cochran D.L., Hoffmann B., Lussi A., Steinemann S.G. (2004). Enhanced bone apposition to a chemically modified SLA titanium surface. J. Dent. Res..

[20] Zhao G., Schwartz Z., Wieland M., Rupp F., Geis-Gerstorfer J., Cochran D.L., Boyan B.D. (2005). High surface energy enhances cell response to titanium substrate microstructure. J. Biomed. Mater. Res. A.

[21] Wennerberg A., Albrektsson T. (2009). Effects of titanium surface topography on bone integration: A systematic review. Clin. Oral Implants Res..

[22] Nishimura T., Ogino Y., Ayukawa Y., Koyano K. (2018). Influence of the wettability of different titanium surface topographies on initial cellular behavior. Dent. Mater. J..

[23] Suchünemann F.H., Galárraga-Vinueza M.E., Magini R., Fredel M., Silva F., Souza J.C.M., Zhang Y., Henriques B. (2019). Zirconia surface modifications for implant dentistry. Mater. Sci. Eng. C.

[24] Gittens R.A., Olivares-Navarrete R., Cheng A., Anderson D.M., McLachlan T., Stephan I., Geis-Gerstorfer J., Sandhage K.H., Fedorov A.G., Rupp F. (2013). The roles of titanium surface micro/nanotopography and wettability on the differential response of human osteoblast lineage cells. Acta Biomater..

[25] Jakovac M., Klaser T., Bafti A., Skoko Z., Pavić L., Žic M. (2022). The effect of Y^3+^ addition on morphology, structure, and electrical properties of yttria-stabilized tetragonal zirconia dental materials. Materials.

[26] Kim M.-H., Park K., Choi K.-H., Kim S.-H., Kim S.E., Jeong C.-M., Huh J.-B. (2015). Cell adhesion and in vivo osseointegration of sandblasted/acid etched/anodized dental implants. Int. J. Mol. Sci..

[27] Borlaf M., Szubra N., Serra-Capdevila A., Kubiak W.W., Graule T. (2020). Fabrication of ZrO_2_ and ATZ materials via UV-LCM-DLP additive manufacturing technology. J. Eur. Ceram. Soc..

[28] Hirano T., Sasaki H., Honma S., Furuya Y., Miura T., Yajima Y., Yoshinari M. (2015). Proliferation and osteogenic differentiation of human mesenchymal stem cells on zirconia and titanium with different surface topography. Dent. Mater. J..

[29] Rupp F., Gittens R.A., Scheideler L., Marmur A., Boyan B.D., Schwartz Z., Geis-Gerstorfer J. (2014). A review on the wettability of dental implant surfaces I: Theoretical and experimental aspects. Acta Biomater..

[30] Uchida M., Kim H.-M., Kokubo T., Tanaka K., Nakamura T. (2002). Structural dependence of apatite formation on zirconia gels in a simulated body fluid. J. Ceram. Soc. Jpn..

[31] Li Q., Li C., Wang Y. (2022). Effect of femtosecond laser ablate ultra-fine microgrooves on surface properties of dental zirconia materials. J. Mech. Behav. Biomed. Mater..

[32] Zhang F., Spies B.C., Willems E., Inokoshi M., Wesemann C., Cokic S.M., Hache B., Kohal R.J., Altmann B., Vleugels J. (2022). 3D printed zirconia dental implants with integrated directional surface pores combine mechanical strength with favorable osteoblast response. Acta Biomater..

